# Change in Stress and Positive Experiences among Older Oregonians during the COVID-19 Pandemic

**DOI:** 10.1093/geroni/igab046.2675

**Published:** 2021-12-17

**Authors:** Maria Kurth, Hye Soo Lee, Soyoung Choun, Carolyn Aldwin, Dylan Lee

**Affiliations:** Oregon State University, Corvallis, Oregon, United States

## Abstract

Several cross-sectional studies have examined stressors and positive events among older adults during COVID-19. We extend these studies by examining changes across time in perceptions of stress and positive experiences. Older adults in Oregon (Mage = 71.1, SD = 7.3, range = 51-95) completed weekly surveys from April 28 to June 23, responding to an adaptation of the Daily Stress Inventory (DISE; Almeida et al., 2002). DISE examines stressors and positive experiences across six domains (health, spouse/partner, other relationships, work/volunteer, finances, and retirement) on a 7-point scale (1 = not at all to 7 = extremely). At baseline, those who felt more stressed were younger, female, and reported more chronic health conditions, while younger adults, especially males, reported more positive events. Positive and stress intensity scores were not correlated. Multilevel models found that for both positive, Blinear = -2.54, SE = 0.52, p < .001; Bquadratic = 0.21, p < .05, and stress, Blinear = -0.79, p < .001; Bquadratic = 0.07, p <.01, intensity showed decelerated decreases across time; residuals for both models were significant. Older adults had lower stress levels, while women and those with chronic health conditions had higher stress levels. Women also reported lower levels of positive events. In both models, neither age, gender, nor chronic health conditions predicted change. These results highlight the evolving experiences during COVID-19, as perceptions of stress and positive events decreased. Future studies should examine how the changing circumstances during COVID-19 affect adaptation, including perceived stress and positive events.

